# Cycling through 360° Virtual Reality Tourism for Senior Citizens: Empirical Analysis of an Assistive Technology

**DOI:** 10.3390/s22166169

**Published:** 2022-08-17

**Authors:** Cláudia Pedro Ortet, Ana Isabel Veloso, Liliana Vale Costa

**Affiliations:** Department of Communication and Art—DigiMedia, University of Aveiro, 3810-193 Aveiro, Portugal

**Keywords:** virtual reality, 360°, head-mounted displays, assistive technologies, cyclotourism, senior citizens

## Abstract

In recent years, there has been a renewed interest in using virtual reality (VR) to (re)create different scenarios and environments with interactive and immersive experiences. Although VR has been popular in the tourism sector to reconfigure tourists’ relationships with places and overcome mobility restrictions, its usage in senior cyclotourism has been understudied. VR is suggested to positively impact tourism promotion, cycling simulation, and active and healthy ageing due to physical and mental rehabilitation. The purpose of this study is to assess the senior citizens’ perceived experience and attitudes toward a designed 360° VR cyclotouristic experiment, using a head-mounted display (HMD) setting within a laboratory context. A total of 76 participants aged between 50 and 97 years old were involved in convergent parallel mixed-method research, and data were collected using a questionnaire based on the technology acceptance model, as well as the researchers’ field notes. Findings suggest that 360° VR with HMD can be an effective assistive technology to foster senior cyclotourism by promoting tourism sites, simulating the cycling pedaling effect, and improving senior citizens’ general wellbeing and independence with physical and mental rehabilitation.

## 1. Introduction

Possible age-associated impairments (e.g., deficiencies in the central nervous and/or sensory physiological systems) [1], alongside the popularity of assistive technologies [2], and senior citizens’ right to leisure and recreation [3], lately challenged by the COVID-19 pandemic, have heightened the importance of finding alternatives to traveling and senior tourism.

Indeed, the COVID-19 outbreak affected various sectors with health spending and employment [4], and travel and mobility tourism constrictions [5] were likely to negatively impact senior citizens’ lives due to the distancing and lockdown measures (e.g., social isolation, cognitive impairments) [6,7]. In this sense, assistive technologies that aim to increase, maintain, or improve the functional capabilities of individuals with limitations [8,9] have been fundamental to people with chronic conditions and disabilities [10,11].

Although several studies on immersive reality technologies have demonstrated to be successful in promoting tourism [12,13] and fostering senior citizens’ wellbeing [14,15], its use in cyclotourism has been overlooked. Even though there has been some research on cycling for senior citizens [16], 360° virtual reality (VR) applied as an assistive technology for senior cycling in a leisure context seems to be unexplored in the scholarly literature. 

VR can be described as an immersive and interactive digital experience, aiming to simulate the physical or fictional contexts [17] in diverse contexts such as mental and physical therapy [18,19] and tourism [20]. This technology sounds promising given the use of panoramic, co-presence effects, interactions with virtual objects, and possible implications [21] that these may present to the pre-touristic experience, touristic experience, and post-touristic experience. Moreover, the increasing interest in virtual tourism [22], sense of ‘staycation’ [23], and fictional traveling [24] exacerbated by the COVID-19 pandemic have become central to reinvesting in tourism promotion strategies and promoting active and healthy ageing. 

The purpose of this study is to assess senior citizens’ perceived experience and attitudes toward a designed 360° VR cyclotouristic experience, using a head-mounted display (HMD) setting within a laboratory context, by answering the research question: “How can 360° VR foster senior citizens’ cyclotourism experience?”. The following hypotheses were anticipated: (a) there are differences in younger and older senior citizens concerning the promotion of the touristic experience; (b) social interaction and safety confidence in VR activities are key for senior citizens to have fun. This confidence is often encouraged by family, friends, and caregivers. These hypotheses are supported in (i) Moniz and colleagues’ study [25], in which the authors found that younger senior citizens, aged between 55 and 64 years old, tend to be tourists who not only value learning about the destination and themselves, but also give importance to social interactions and fun in comparison with older age cohorts; and in (ii) Barsaella and colleagues’ research [26], stating that senior citizens highlighted the importance of the family, friends and caregivers’ influence in assuring that VR is safe and fun to engage with the activity and foster social interactions. As such, convergent parallel mixed-method research involving both qualitative and quantitative approaches was followed, using a questionnaire based on the technology acceptance model [26], and the researcher’s field notes as instruments for data collection.

This paper is structured as follows: Section 2 explores the use of VR and its potential benefits by describing related work on senior citizens, tourism, and cycling. Section 3 covers the experiment method, participants’ characterization, data collection, analysis procedures, and ethical issues. Section 4 reports the main results and findings, whereas Section 5 is devoted to its discussion. Finally, Section 6 ends with some conclusions, limitations, and future research directions.

## 2. Background Research

VR is often associated with sensory immersion (e.g., auditory, visual, and tactile sensors), interaction, and a sense of ‘being there’ [27], in which users can interact with a computational interface. Virtual, augmented, and mixed realities represent computational interface techniques that consider the three-dimensional (3D) space and enable users to explore through their senses. VR depends on visualization equipment (i.e., monitors, projectors, helmets, and glasses), whereas augmented reality (AR) does not present such restrictions because it enables the overlay, composition, and visualization of virtual objects in physical-world environments [28]. Moreover, mixed realities (MRs) integrate both physical and digital environments by encompassing (i) virtual environments, (ii) virtual reality, (iii) augmented reality, and (iv) physical environments [29], i.e., MR unites features of VR with AR by inserting virtual objects into the physical world and enabling user interactions, producing new environments in which physical and virtual objects coexist in real time.

According to LaViola and colleagues [30], considering some criteria when classifying VR input and output devices is essential. In terms of input devices, one may enlist the following features: (i) degrees of freedom, i.e., the number of dimensions with which the system interacts; (ii) ergonomics, i.e., the level of comfort felt by the user; (iii) frequency of data sent to the system, because this is related to the delays between the user action and the system response; (iv) feedback; and (v) the type of task [30]. 

In terms of output devices, these have the following parameters: (i) field of view (environment’s angular extent observed in a certain time); (ii) field of regard (a measure of the view angle); (iii) resolution (a measure of the image quality); (iv) screen geometry, i.e., rectangular, hemispherical, or hybrid; (v) light transfer by front and rear projection (e.g., projectors), or laser projected onto retina; (vi) update rate (i.e., measure in hertz of the image’s refresh rate); and (v) ergonomics [30].

Output devices can be divided into three groups. The first group is visual devices (e.g., screens, projectors, HMD, and holograms) [31], which are based on the quality of the images generated, being greatly influenced by the perception of the immersion level of a VR system. The second group is auditory devices, in which the sound is intended to provide a feeling of immersion. Finally, the third group is physical devices, which aim to stimulate physical sensations (e.g., touch, temperature, and muscle tension). 

Furthermore, VR can englobe three factors: the virtual space, presence, and entertainment [32]. Following the McLuhan’s notion of involvement [33], the virtual space can be defined as a virtual environment in which real experiences happen. Such a notion is, nowadays, often portrayed as a form of human–computer interaction (HCI) in which users have interactable experiences with the aid of 3D graphics, noticeable in the concept of presence [34] in artificial computer-generated environments [35]. Secondly, presence is what acknowledges people to perceive the world around them, and when mediated by technology, users can recognize the unmediated (i.e., presence) and the mediated (i.e., telepresence) environment [36]. In other words, sensing the virtual space as if being there is important [35]. Thirdly, VR experiences are usually linked to the degree of entertainment which is aligned with human factors, because it can trigger physiological (e.g., sensorimotor) and psychological (e.g., motivational and emotional) responses [37,38], as studied in the field of cognitive infocommunication (CogInfoCom) [39].

VR often uses virtual scenarios that enable the user to move freely in the virtual world, whereas 360° VR emerges by offering spherical panoramic experiences. Spherical/immersive 360° video [40] has been popular in the contemporary audiovisual panorama due to scenario believability. Since 360° VR content relies on real-world photos and videos, it tends to be more representative of the intended environment [41], allowing the user to have a clearer detailed view and enjoyable experience [42]. Wu and Lin [43] consider that virtual reality environments are directly linked to 360° videos, impacting the perceived sense of presence and entertainment [44].

Following this analysis, HMDs are VR output devices considered entirely immersive wearable instruments that provide a full field of view that enables complete isolation of the user from the physical world (i.e., create more visual realism). An HMD, or real 3D immersive VR, is composed of a modulated light source visualized through an optical system (e.g., liquid-crystal displays) which is worn on the user’s head through a band, helmet, or around an eyeglass frame. Immersive HMDs are systems in which the optics are positioned in front of the eyes, blocking most of the visual field [45]. In short, HMD have (i) large, wide-ranging screens; (ii) miniaturization and weight reduction (i.e., usability); (iii) utility; (iv) interactive spatial information; and (v) images superimposed by a see-through function [45].

However, it is important to recognize the limitations of VR and HMD, such as discomfort, nausea, cost of equipment, and unfamiliarity. In fact, two components are essential to apply VR and overcome the mentioned limitations. These are: (i) creating a virtual world through the video capturing or 3D modeling; and (ii) experimenting with it using a device in which users can immerse themselves in the virtual environment.

In the context of this research, a 360° VR cyclotourism experience was developed with the target audience of senior citizens. In this section, a brief overview of the related research on VR applied to tourism, cycling, and senior citizens is provided.

### 2.1. VR in Tourism

The tourism industry has been undergoing significant changes resulting from technological advances and increases in the production of online content, which tends to influence consumer behavior [46]. Contemporary tourists tend to be more knowledgeable, demanding, cultured, and may be exhausted of the traditional offers, abandoning passivity to become more dynamic and participative [47]. They also tend to be changing their motivations, needs, and desires, reflected in their demand for tourism offers, as they intend to live unprecedented experiences, and in which origin (i.e., from reality or fiction) seems to be no longer one of the major concerns. Virtual (reality) tourism is likely to meet that context, in which a technological environment (e.g., 3D technology that immerse participants in the virtual environment) can bring liveliness to alternative virtual experiences [48].

Whether in a pandemic context or not, VR in tourism has benefited both the business and destination spectrum and customers [20]. For example, research has evidenced profits and outcomes in these aspects:

- Sustainability and heritage preservation to diminish overtourism and act as an alternative to visiting protected/preserved natural sites [49], or even as being a low-cost and sustainable way of traveling;
- Travel planning and management [50], because when exploring a place before purchasing, tourists can build a list of the various locations they want to know/visit, becoming more informed about the destination, and avoiding disappointment during the visit;
- Marketing promotion and sales [51], which can be valuable for destinations that do not have a famous attraction or a well-recognized name, but with great natural and cultural attributes;
- Enhanced experiences [52], given the opportunity to obtain in-depth information about a specific place;
- Immersion, entertainment, and engagement [53,54], because sensation amplifies the believability of the scenario with which users are dealing;
- Social interactions and connectivity, through the possibility to encounter or meet users and interact with them [55], among others.

According to Beck and colleagues [56], a virtual environment within a VR tourism context involves an efficient non-, quasi-, or fully immersive VR system with artificial content or 360° videos. Such may facilitate virtual touristic experiences while stimulating the senses—especially the visual sense—to potentiate the entertainment, inheritance protection, scheduling, management, information exchange, promotion, education, and accessibility before, during, or after traveling [56]. 

Although VR in tourism contexts may be widely available, some barriers in terms of access (e.g., lack of smartphone and computer, absence, or poor internet connection) may compromise its usage and popularity (i.e., people choose not to use it). Although VR tourism does not replace physical travel, it democratizes an activity that overcomes spatial and temporal barriers, extending the traveling experience regardless of financial, physical, and psychological conditions.

### 2.2. VR in Cycling

Riding a bicycle may bring several benefits concerning health and environmental sustainability [57], but also shortcomings in bike fitting (e.g., saddle too high) and risk of accidents, which may lead to injuries. Disease prevention may be highlighted as a health benefit because the cycling activity stimulates the cardiopulmonary and muscular system, preventing and delaying the onset of various diseases such as diabetes, high cholesterol, acute myocardial infarction, and stroke. It is worth acknowledging that although cycling contributes to a healthy lifestyle, it does not cure or stop individuals from having the abovementioned diseases. Anxiety and stress may also be reduced when pedaling, given that the body stimulates the release of endorphins which promote relaxation, and serotonin which helps to maintain a good mood; aids in motor coordination; tones muscles and strengthens joints, which prevents arthritis; among others [58,59]. It also reinforces social values such as companionship, respect, tolerance, and the feeling of belonging. Concerning the environment, cycling contributes to zero CO_2_ emissions, avoiding greenhouse gas and other fossil fuel components. Riding a bicycle also reduces traffic jams and noise pollution [60,61].

Cycling is not always possible in the conventional way (i.e., cycling outdoors), and indoor virtual cycling is, therefore, a solution to this necessity. Virtual cycling or cycling simulation has reached its popularity boom with the emergence of ‘smart’ training rollers and programs such as Zwift (https://www.zwift.com/eu, accessed on 16 March 2022). Cycling indoors offers several advantages such as safety and control of training, regardless of weather conditions, representing a viable and realistic alternative for amateurs and professionals.

According to Sun and Qing [62], cycling simulation embodies three general categories. These are:

- Replica or the replication of the cyclist effort, in which the bicycle dynamics guide the cyclist’s movement with tactile feedback [63,64];
- Rehab involving the application of medical rehabilitation and therapy [65,66];
- Behavior involving the research of human factors and behaviors in cycling [67,68].

In addition, Sun and Qing mentioned the existence of commercial systems aimed at fitness and training, including the incorporation of racing videos or famous routes such as the Tour de France [62].

The applicability of virtual cycling simulation has several forms of conducting the research experiment. Namely, it is possible to simulate in (i) a simpler way, with the use of stationary bicycles or bikes mounted in rollers, and VR equipment; or in (ii) a complex way, by using high-end hardware and software engine and coding. Regarding hardware, it is common to use bicycle simulators with stands or motion platforms to mimic physical forces (e.g., speed and power), trepidation (i.e., bicycle reaction to pavement types), freedom of movement (e.g., turning and braking), and slope. Through software, it is possible to provide a visually appealing graphic scenario, calibration, and its validity (i.e., close to reality).

Similarly to other applications of VR, using it in cycling may also have some limitations, e.g., the distant relation to the actual outdoor cycling feeling in terms of physical effort, surroundings, and landscape.

### 2.3. Senior Citizens Using VR

The exponential growth in the ageing population paralleled to other age groups is noticeable through the inverted demographic pyramid. In fact, senior citizens often represent a susceptible group at risk of social and digital marginalization [69], sedentary lifestyle, agility limitations, health scarcities, and mortality [70]. In later life, physical changes are likely to arise in global sensory impairment, balance, and the control of movements [71], and decline in function of vital organs (e.g., heart, lungs and kidneys, muscle mass, hormone levels and brain) [72]. Physical ageing may be more straightforwardly evident; therefore, the psychological effects sometimes have a higher impact [73]. Deficits in attentional capacities and memory are often intertwined to cognitive decline, depression, and social isolation [74,75].

Such impairments give rise to the possibility of intervention, assessment, and planning of projects and activities for senior citizens, aimed at preventing difficulties related to cognitive decline, as well as therapy. Assistive technologies rely on resources (e.g., products, equipment, hardware, and software) and services (i.e., assistance, evaluations, testing, and training for using the resources) used with the aim of increasing, maintaining, or improving the functional capabilities of people with disabilities [76]. Moreover, these can be customizable (adaptative) to attend to the individuals’ cognitive, emotional, social, and physical state. In detail, cognitive assistive technologies can train cognition, logic, creativity, and learning challenges. As for the emotional state, the impact of sound or visual effects on emotions, sense of empathy, and emotional and visceral engagement, can be fulfilled. Social-based challenges, self-expression, and communication tend to also be a concern. Finally, the users’ physical conditions must also be attended, creating goals that suit their context [76].

In the same logic, the use of VR with wearable devices, such as HMD, is suggested to be quite effective in the rehabilitation of senior citizens with physical or cognitive disabilities (e.g., attention, executive functions, memory, motor recovery, special abilities, and spatial orientation). VR can create a motivating environment for learning and understanding a user’s perceptual and motor skills’ characteristics [77,78]. Furthermore, VR and HMD are tools that healthcare professionals and caregivers may adopt. It is easier for senior citizens to become involved in virtual reality than physical activities [79]; therefore, they can exercise their mind and body independently and regularly, improving physical, cognitive, and psychosocial functions for their general wellbeing.

According to Campelo and colleagues [80], one of the benefits of using VR is the increase in rehabilitation time at a lower cost when compared with conventional programs, allowing the therapist to provide a greater variety of stimuli with greater specificity compared with traditional methods. In fact, the use of VR with senior citizens has shown positive results in several areas: (i) in the physical field, controlling posture and balance, gait speed, range of motion and energy expenditure was possible; (ii) in terms of cognition, executive function, attention, and memory improved; and (iii) in terms of the psychosocial domain, improvements in symptoms of depression, anxiety, mood, and social interaction were observed [80].

Campelo and colleagues also developed a model entitled VRehab [80], for the application of virtual rehabilitation with senior citizens divided into four areas: (i) beneficiaries and providers, (ii) virtual environments, (iii) social engagement, and (iv) healthcare application. The system’s beneficiaries and providers are senior citizens, health care providers, researchers, and VR designers. Virtual environments may include 3D tracking systems, augmented reality systems, cave automatic virtual environment (CAVE), exergames, haptic interfaces, and simulators. Social engagement depends on VR accessibility, attitude change, costs, cultural sensitivity, and safety concerns. Furthermore, the applications of virtual reality in health include physical rehabilitation, cognitive training, psychosocial rehabilitation, surgery simulations, and physical literacy development.

However, there are some limitations given that this audience tends to be unfamiliar with the technology and may be hesitant in accepting its usage, because sometimes they may not comprehend its possible contributions. Thus, a technology acceptance model [26] was used in this study because it can explain the senior citizens’ acceptance of VR based on their behavioral intention and determine users’ perception of usefulness and easiness, as described in the following section.

## 3. Methodology

The purpose of this research was to assess the senior citizens’ perceived experience and attitudes toward a designed 360° VR cyclotouristic experiment, using the HMD setting within a laboratory context, by answering the research question “How can 360° VR foster cyclotourism experience?”. Additionally, the following hypotheses were anticipated: (a) there are differences in younger and older senior citizens concerning the promotion of the touristic experience; (b) social interaction and safety confidence in VR activities are key for senior citizens to have fun, being encouraged by family, friends, and caregivers. With this experiment, it is also intended that participants evaluate the experience, giving their perceptions and opinions about using VR in a cyclotourism context.

**Figure 1:**
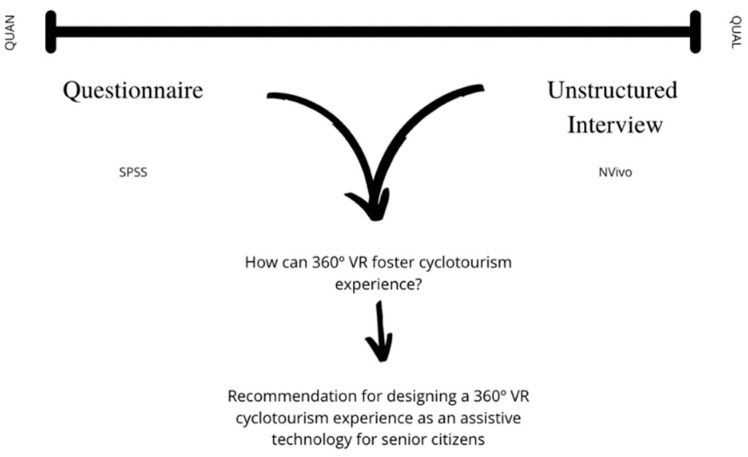
Schematic overview of the convergent parallel mixed-method approach.

The use of qualitative and quantitative methods was needed; thus, a convergent parallel mixed-method approach was followed within a laboratory setting, as depicted in Figure 1. 

A virtual cyclotourism activity, entitled “Senior Virtual Reality Meeting: A Trip to Paris” was conducted from 20 October to 17 November 2021, with 14 sessions of about one and a half hour each, in the Department of Communication and Art at the University of Aveiro, Portugal. This activity was conducted due to the request of institutions of the Third Age to provide an experience with VR technology for senior citizens. Subsequently, the researchers planned and developed a cyclotouristic trip to Paris, because it appealed to the participants. Such choice was based on the city’s fame and its monuments, but also the pandemic situation that inhibited the participants from traveling (due to the restrictions imposed) and from having moments of conviviality and sharing.

The following sections clarify the experimental process, the technology acceptance- based questionnaire, unstructured interview and data analysis questions, the participants’ recruitment and characterization, and the data collection and analysis procedures, ending with ethical concerns involving the target audience. 

### 3.1. The Experiment

The virtual cyclotourism activity was divided into two phases (Table 1), and each lasted for approximately 3 minutes per participant. This duration was considered the most appropriate for them to become accustomed with the technology, but also to not overload them in terms of receiving visual information different from what they were used to, thus preventing nausea and discomfort (i.e., simulator sickness) [81]. Some participants had been in lockdown since the beginning of the pandemic, which affected them at physical and mental levels reported by themselves and caregivers (e.g., muscle atrophy and depressive symptoms). Thus, the researchers needed to approach all the matters subtly and conveniently, as described subsequently.

While preparing for the activity, a questionnaire about the experience was elaborated, proceeding with a selection of 360° videos and background music, and participants informed consent. The questionnaire was based on the survey from Barsasella and colleagues [26] grounded on the Technology Acceptance Model and the opinion of 30 senior citizens about virtual reality in 12 sessions (15 minutes each for six weeks). In addition, the Kaiser–Meyer–Olkin measure of sampling adequacy and Bartlett’s test was used to ensure the reliability of each question, and adapted to the context of cyclotourism (cf., Section 3.2. The Questionnaire).

Two 360° 4K videos were selected and downloaded from YouTube. The first was a visit to the Eiffel Tower (https://www.youtube.com/watch?v=HNApxhvK1Hg, accessed on 18 October 2021), whereas the second one was a cycling trip by the Parisian suburbs. (https://www.youtube.com/watch?v=tI3bKRRMAKA, accessed: 18 October 2021) Background music based on Parisienne instrumental jazz (https://www.youtube.com/watch?v=tkjlYxUf4-M, accessed: 18 October 2021) was also added to build an immersive environment. Finally, the informed consent safeguarded the participants and/or their caregivers’ acknowledgment of the study, instruments used for data collection, and publication of results.

Regarding the setup, it contained the computer that connected the HMD; the VIVE HMD; a 58 inch/147 cm LED Ultra HD (4K) Smart TV, with HDMI connection to the computer, so that the remaining, generally, five participants (i.e., watchers) could comprehend what the person testing (i.e., tester) was seeing; two cameras for audio and video recording; chairs for the senior citizens that were observing; an armchair for the participant who was testing; a stationary bicycle; and a fan. The setup was arranged so that one researcher could conduct the experiment, while another could observe and take notes of the participants’ reactions. Nevertheless, the two cameras, located at strategic points, collected the audio and video of senior citizens who watched the experience and those who were experiencing it. One of the goals was also the senior citizens’ observation–participation in the whole activity (i.e., phase 1 and phase 2), not only to prevent them from being afraid of testing, but also to encourage social interaction. In detail, the participants arrived in a group of approximately six, and while one participant at a time performed the experiments, the others watched on the Smart TV.

**Figure 2:**
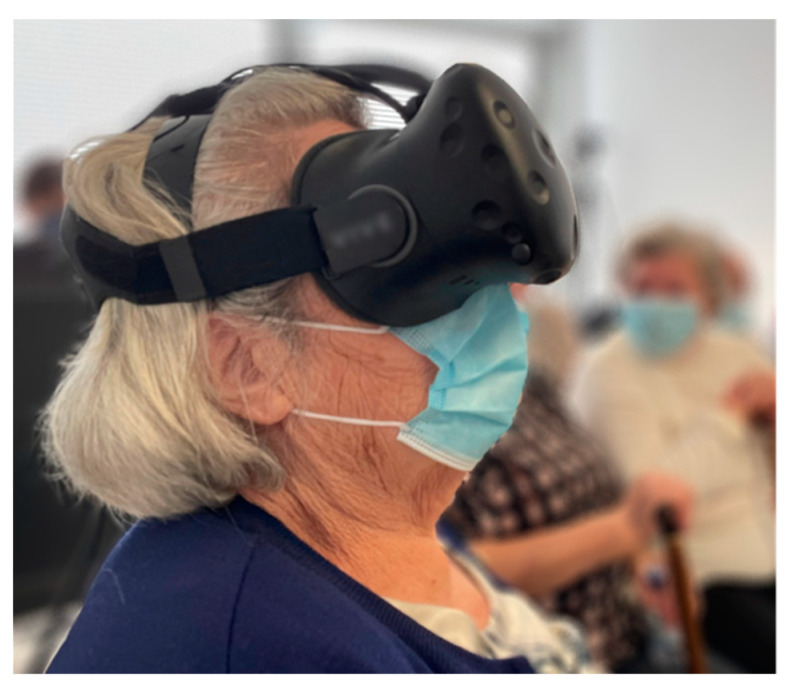
Participant testing the HMD in the first phase of the experiment.

The first phase of the experiment was based on the primary contact with VR, i.e., the adaptation to the use of HMD (Figure 2) in terms of graphic visualization and comfort. It lasted about 3 minutes per participant where, initially, the purpose of the senior citizens’ participation was introduced, explaining VR and how the HMD worked, what they would experience, and what would be evaluated. In this phase, senior citizens were invited, one by one, to sit in the armchair and put on the HMD to familiarize themselves and ascend the Eiffel Tower. While the participants watched the 360° video of the Eiffel Tower (the tester on the HMD and the watchers on the Smart TV), the background music was playing. Participants, both testers and watchers, were dialoguing while describing what they were seeing and creating narratives about the Eiffel Tower, and Gustave Eiffel and Thomas Edison, as their figures appeared on the video.

**Figure 3:**
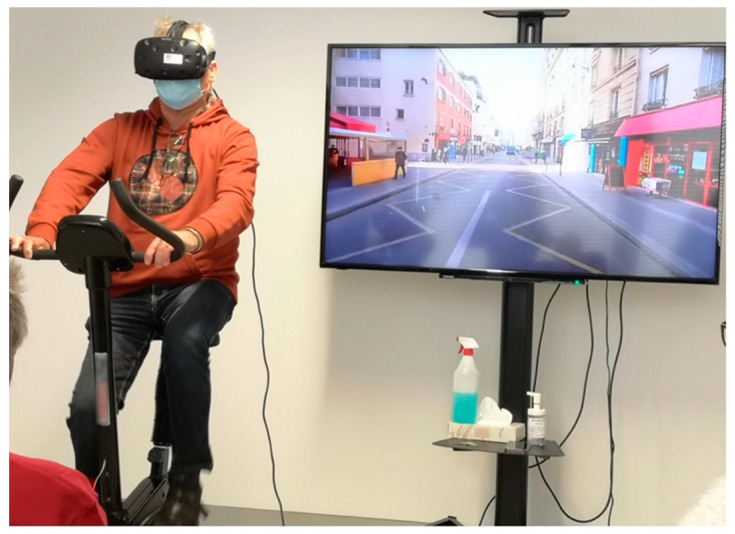
Participant testing the 360° VR cyclotourism experiment (phase two).

The second phase was based on the cyclotourism experience (Figure 3) and lasted an average duration of 3.5 minutes per participant. Here, participants mounted the stationary bicycle and put on the HMD. They were warned that (i) they would only have to pedal to move or stop to stay in place, and (ii) that they could not choose the direction or try to turn the handlebars. Moreover, they were also told about the lack of need to comply with circulation signs, as traffic and pedestrians would not get in their way.

Similar to the first phase, a video of Paris’ suburbs was playing alongside the background music; however, a fan was included for air circulation because participants were putting on effort while pedaling. During this phase of the experiment, most of the participants shared their memories of the streets of Paris (those who had been there), similar cities, and their wishes to visit other places with VR technology. Moreover, they also encouraged each other before and throughout the experiment. Afterwards, all the participants filled out the questionnaire and suggested places for a future experiment.

### 3.2. The Survey Research

A questionnaire (Table 2), based on Barsasella and colleagues’ [26] study, was developed and used to gather the participants’ perspectives on using 360° VR with HMD in cyclotourism to identify the experience as a suitable assistive technology. 

The questionnaire sections included sample characterization (containing questions about age and gender); whether participants ever visited Paris; and satisfaction of use related to the 360° VR experience, as demonstrated in the Data Collection column of Table 2, answered using a 5-point Likert scale, in which 1 was ‘I totally disagree’ and 5 was ‘I totally agree’; and further comments.

Data collection items were simplified so that all participants could understand and respond. Seven out of the thirteen items were adapted from the Barsasella and colleagues’ questionnaire [26], and an additional six items comprised the applied instrument to meet the purpose of the study. In specific, the adopted items were: (i) Q2. ‘*The virtual reality experience was comfortable (e.g., no nausea)’* adapted from *‘VR was comfortable to use’*; (ii) Q3. ‘*I enjoyed using virtual reality technology’* adapted from *‘I enjoy using VR’*; (iii) Q4. ‘*I had fun using virtual reality technology’* adapted from *‘I have fun when I use VR’*; (iv) Q5. ‘*My family and friends influenced me to use virtual reality’* adapted from ‘*My family members think I should use VR* and *People who are friends and acquaintances have influence on my intention to use VR’*; (v) Q6. ‘*My caregivers encouraged me to use virtual reality’* adapted from ‘*People who take care of me encourage me to use VR’*; (vi) Q7. ‘*Virtual reality can be a technology capable of improving my state of mind’* adapted from *‘VR is an efficient tool to raise my mood,’* and (vii) Q8. ‘*In the future, I would like to use virtual reality to relax my mind’* adapted from ‘*In the future, I intend to use the device for mental relaxation*.’ 

The additional questions (Q1, Q9, Q10, Q11, Q12, and Q13) complemented the study insofar as it was important to analyze the ease of being able to see the experience using the HMD (having the point of comparison of what was seen on the Smart TV), if these activities may foster social interactions, as well as if all the data referring to cyclotourism have pertinence in the combination of VR technology with this subject and target audience. 

Additionally, the participants’ caregivers (when applied) were interviewed—following an unstructured interview—to manifest their opinions not only on the overall experiment as assistive technology, but also on the immediate effects on the target audience. In detail, two central guiding questions were asked to all the caregivers based on: (i) “What is your opinion on the 360° VR cyclotouristic experience?” and (ii) “What were the immediate effects of this assistive technology on the participants’ (physical and mental) wellbeing?”, both followed by prompts and probing questions for further elaboration and exposition on the responses. This unstructured interview lasted between 5 and 10 min and allowed the caregivers’ feedback. Moreover, data were collected through field notes, and data analysis resulted in two themes: (i) use of the technology (i.e., technology acceptance), and (ii) effects of the experiment. 

Data collection questions were tested to measure internal consistency and correlation based on the hypothesis of a relation between each item. The data analysis questions were answered with the support of the questionnaire, participant observation, field notes, caregivers’ opinions, and the literature.

### 3.3. The Participants

A convenience sample was recruited by invitation email (Institutions of the Third Age contact researchers to perform an activity with this technology; however, a time span of 2 months was needed to design the experiment, have the ethical concerns approved by the committee of ethics and deontology, invite the institutions, and confirm the availability of days for both parties). 

The sample consisted of 76 senior citizens, with an average age of approximately 74 years old (minimum = 50; maximum = 97; standard deviation = 12.04), 78% females (*n* = 59) and 22% males (*n* = 17), as illustrated in Table 3. The criteria used for the participants’ selection were: (a) being aged 50 years or older—to fit within the identified age cohorts: pre-senior (50–64 years old) [82], young-senior (65–74 years old), middle-senior (75–84 years old), and old-senior (85+ years old) [83], and (b) voluntary participation. None of the participants had previous experience with VR; 39% (*n* = 30) were institutionalized and it was their first outing since the beginning of the pandemic; 7% (*n* = 5) were illiterate; 8% (*n* = 6) had never ridden a bicycle or were afraid of riding one; 51% (*n* = 39) had physical impairments (e.g., partial blindness and glaucoma, prostheses, wheelchair, assistive cane, and walker users); and finally, 72% (*n* = 55) had never been in Paris.

The sample ensured heterogeneity of participants due to the randomness of this experimental research. It is believed that different characteristics reinforce the results and findings because it is easy to assume that a participant who has never ridden a bicycle or is afraid of it will compromise (negatively) the outcomes. However, there was not such an influence on the experiment. Although gender may not be adequately represented in this study and there may be some member and gender bias, because some groups of participants were from the same institution, the researchers ensured that all participants expressed their opinions with the data collected from the questionnaire, the interaction between participants, researchers, and caregivers. In detail, the importance of ensuring individual opinions is because opinions may be influenced by the group, not reflecting what each participant felt about the experiment.

Additionally, 15 caregivers (4 psychologists, 4 gerontologists, and 7 care assistants) were interviewed (following a non-structured interview). 

### 3.4. Data Collection and Analysis Procedures

Data were collected from 20 October to 17 November 2021 in 14 experiment sessions that were conducted with an average of 5 to 6 participants and at least 1 caregiver per session, as previously explained. Data triangulation from the different data sources (i.e., questionnaire, field notes, and unstructured interview) was performed to assure the validity of the research. 

After collecting the data, the participants’ perspectives and caregivers’ feedback gathered from the ongoing experiment (i.e., field notes, and video and audio records) were divided into words, phrases, or sentences using open coding to be compiled in different categories according to the context and number of occurrences. Furthermore, the participants’ questionnaire responses were divided into variables. 

Data analysis was performed using IBM SPSS Statistics 28.0.1.0 (https://www.ibm.com/analytics/spss-statistics-software (15 January 2022).) and NVivo 1.5.1 (https://www.qsrinternational.com/nvivo-qualitative-data-analysis-software/home (15 January 2022)). To ensure code reliability, coding in each software was reviewed by the researchers of this study, so that the results could be more accurate. Regarding the quantitative approach, every statement value was coded (e.g., Female = 1 and Male = 2). Furthermore, SPSS tests (i.e., Cronbach’s alfa and Pearson’s correlation) were performed to safeguard the data collection consistency.

Moreover, findings from the participants’ observation, opinions and reactions, and caregivers’ feedback were also collected and analyzed to cross with the literature and the results, thus supporting the outcomes.

### 3.5. Ethical Concerns

This study adhered to the Ethics and Deontology Council of the University of Aveiro Ethical Approval for the SEDUCE 2.0 research project POCI-01-0145-FEDER-031696 (SEDUCE 2.0—Senior Citizen Use of Communication and Information in miOne community) and doctoral program in Information and Communication in Digital Platforms (University of Aveiro and University of Porto) under the FCT (Fundação para a Ciência e Tecnologia) grant nr. 2020.04815.BD. Furthermore, it safeguarded: (a) the informed consent of the participants and their caregivers; (b) voluntary participation; (c) involvement of the research team in the process; (d) that the risks of participating in the study did not outweigh the risks associated with the participants’ wellbeing, and authorization of the recording and capture of images and audio for research purposes.

To reassure voluntary participation, participants and/or their caregivers were given an informed consent form, which communicated the circumstances under which the study was carried out, the purpose of the research, potential risks and benefits, and the right to withdraw at any time and to refuse to answer any question. Researchers assured that all participants and/or their caregivers understood the terms of any agreement before taking part in the research project.

Furthermore, the risks of participating in the study did not outweigh the risks associated with the participants’ daily lives, and the experimental procedures were closely followed and supervised by the research team and participants’ caregivers (when applied). Researchers also assured the confidentiality and the privacy of the data, because all participants were randomly identified with a P and a number, as well as ensured that the private information was not released elsewhere. When reporting the results, only the relevant information to the purpose of the study was written.

## 4. Results and Findings

Crossing data obtained from the results of each method had the purpose of answering the data analysis questions (Table 2) to evaluate the suitability of the experiment in terms of assistive technology. In general terms, the results observed in Table 4 accounted for a positive experience in all items, exceeding expectations, especially considering the different age-cohorts and the participant’s current situation (e.g., (non-) institutionalized; cycling practitioner, or with physical impairments). 

It is worth mentioning that Q5, *My family and friends influenced me to use virtual reality*, and Q6, *My caregivers encouraged me to use virtual reality*, did not account many answers because these either did not apply to all the participants or the participants refused to answer. More specifically, 4% (*n* = 3) did not answer Q5, and 9% (*n* = 7) did not answer Q6. The percentages estimated for the total of participants were registered (*n* = 76), and the levels of completeness and internal consistency were measured using Cronbach’s alfa test with the level of completeness of 87% and reliability of 0.553. When performing the same test without the most unanswered item, Q6, mismatching participant’s context, the completeness raised to 95% and the reliability increased to 0.822.

Findings suggest that 92% of the participants (*n* = 70) totally agreed that the virtual reality experience was easy to see (Q1), and only 4% (*n* = 3) considered it somewhat difficult or very difficult. These results answer the question *What are the main aspects that affect participants’ condition of vision?*, because visual impairments and lens fogging were an observed cause that interfered, mostly due to the use of masks for COVID-19 protection. Nevertheless, the HMD seems to be a wearable device capable of providing ‘easy-to-see’ images and video for most participants (who tend to have a decline in visual capabilities).

Regarding Q2, 93% (*n* = 71) agreed or totally agreed that the experience was comfortable with no nausea (i.e., the experience of seeing the images wearing the HMD goggles in different phases of the experiment). Nevertheless, the remaining 7% (*n* = 5) did not find it natural, causing sickness or dizziness. Answers to the question *What are the main aspects that influence participants’ discomfort and/or sickness?* were found to be, essentially, the smothering sensation when using the HMD with a mask, vertigo syndrome (identified in some participants), and differences in light intensity.

Although all the participants enjoyed using the VR technology (Q3), 93% (*n* = 71) totally agreed that it was a fun technology (Q4). The remaining 7% (*n* = 5) agreed that it was fun. According to the participants’ responses on the questionnaire, it was possible to consider VR an entertainment tool (answer to *Is VR a useful tool for entertainment?*), also being grounded with the following opinions:

*“It’s even hard to go back to the real world. It’s really cool.”*

—P5

*“Worth it (…) I really felt like I was there.”*

—P39

*“I’m ready for many more experiences like this.”*

—P72

Moreover, it was possible to show that most of the participants were influenced by their family and friends (88%, *n* = 67) and/or their caregivers (87%, *n* = 66) to participate in the VR experiment (Q5 and Q6). Participants reckoned on the ability of VR to their self-wellbeing state, because 6% (*n* = 4) would not like to use it to improve their state of mind (Q8), and 8% (*n* = 6) did not think the technology was capable of it (Q7).

Regarding *Which are the main features that a VR experience should have to be closed to tourism?*, the proximity to reality and immersion seems to be the key. The majority of participants (99%, *n* = 75) found VR to be a good way of doing tourism (Q9), because it was closely linked to their real tourist experience, as stated in the following sentences:

*“When I left I was even surprised to be in the room, it felt like I was there.”*

—P2

*“That’s exactly how you see the tower when you’re there. I want to go back there.”*

—P27

*“Just now I was in Paris and I’m already here.”*

—P54

To answer *What are the main factors of using VR in cyclotourism that contribute to active and healthy ageing?*, participants’ opinions on VR and cyclotourism to experience places in a safe way (Q10) and exercise (Q11), were gathered. Most (95%) of the participants (*n* = 72) agreed or totally agreed that combining VR with cyclotourism is safer than the actual cyclotourism activity; however, 3% (*n* = 2) stated that it was not a good form of exercise. Nevertheless, most of the participants’ opinions were similar to the following quotes:

*“Oh cool… and I take the opportunity to exercise.”*

—P10

*“I don’t even like riding a bike, but this one is much more interesting.”*

—P16

*“It would be nice to have this at my home, it would be the perfect way to do some gymnastics. What a spin!”*

—P21

*“Being able to do this is very good for self-esteem.”*

—P45

*“I haven’t ridden a bike for 3 years. Really enjoyed.”*

—P61

*“This is good for exercising and physical therapy, it’s good for the legs.”*

—P62

*“I used to ride my bicycle a lot, it was good.”*

—P75

Regarding *Can VR enhance social interactions?*, all the participants considered the whole experience to be a good way of fostering social interactions (Q12), whereas 7% (*n* = 5) did not feel that they wanted to share it with their family and friends (Q13). Most senior citizens had an encouraging attitude towards their group by sharing their experience when using the HMD and cheering while watching what others were seeing with the HMD on the Smart TV. Additionally, the majority of the participants tended to narrate or tell stories while they were experimenting.

Overall, the experiment has benefited both the research and senior citizens’ wellbeing. Using 360° VR to perform cyclotourism was found to be suitable, because participants considered it easy and comfortable to see, closely linked to the tourism environment, and a secure way to visit places and exercise as a form of physical therapy or physical maintenance. Furthermore, the experiment contributed to social interactions, namely, through encouragement, sharing of the experience with friends and family, and storytelling.

**Figure 4:**
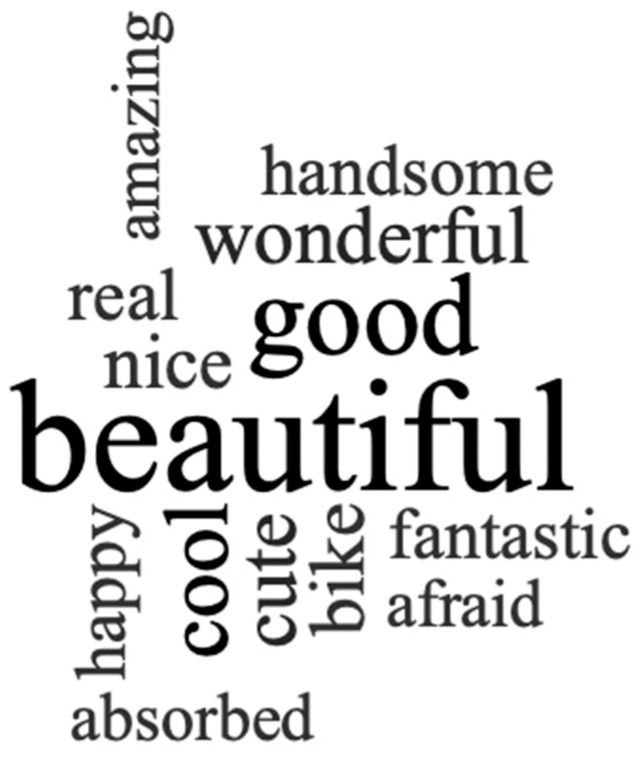
Word frequency of participants’ opinion on the experiment.

Based on the participants’ opinion of the global experience—collected with field notes—the NVivo word frequency tool was utilized to determine whether it was a positive experience. As Figure 4 illustrates, participants considered the experience and the 360° videos mostly beautiful (30 citations), good (15 citations), and cool (8 citations).

A Pearson’s correlation test was performed to observe if there was any linear relationship between the items from the questionnaire. Table 5 shows the significant correlations between the independent variable of age and the questions, whereas Table 6 depicts all significant correlation values between each question, considering that r is the Pearson’s Correlation test results, * *p* < 0.05 and ** *p* < 0.01. Interestingly, no relationship between having visited Paris and the questions of the VR experience was found. In contrast to possible speculation ascertaining that having been to Paris and already knowing the city could either negatively influence—because the participants could consider the experience far from reality—or positively influence—because they had the opportunity to revisit something they already knew, no relationship was verified. 

Moreover, some differences concerning some items were found, with particular emphasis on the influence of family and friends in convincing older participants to try VR (Q5), as well as the older senior citizens feeling that the VR activities were an experience to share (Q13).

Regarding the correlation between the questionnaire’s items, a perfect positive relationship (*r* = 1.000) was found with the influence of family and friends on senior citizens’ participation in the activity (Q5) and the encouragement of their caregivers (Q6). Even though Table 6 demonstrates several significant positive relations, it is important to highlight the following high correlations (shaded in Table 6) found in this study: (i) the enjoyment (Q3) and fun (Q4) of using VR are strongly linked to each other; (ii) as participants had more fun (Q4), they found VR beneficial for tourism (Q9), and also considered the 360° VR cyclotouristic experience to be a good time for socializing (Q12); (iii) the family and friends influence (Q5) is positively associated with the perception of safety in using VR to exercise (Q11); (iv) in the same vein, the influences of family, friends, and caregivers (Q5, Q6) are strongly related with the participants’ willing to share with them (Q13); (v) the senior citizens’ desire to use VR in the future (Q8) is optimistically inter-related with the perception of the suitability of the technology to conduct tourism (Q9), to exercise in a safe way (Q11), and to share with family and friends (Q13); (vii) moreover, when considering VR beneficial to conduct tourism (Q9), there is also an underlying relationship with the combination of the bicycle (i.e., cyclotourism) as not only being a good way to experience places and exercise securely (Q10, Q11), but also to socialize (Q12) and share (Q13); and finally, (viii) senior citizens feel the need to share (Q13) that VR with bicycle is a safe way to exercise (Q11).

Additionally, most of the institutionalized participants’ caregivers (e.g., psychologists, gerontologists, and care assistants) shared their personal opinion on the overall experiment and its effects on the participants. When surveyed about the theme of technology use, they were impressed with the acceptance of the experiment by those psychologically affected (e.g., by depression and dementia), the more sedentary participants, and those with physical limitations. Such may be justified by the participation and encouragement of the group, but also by being able to watch what the participants were experiencing. Secondly, in the scope of the effects of the experiment, the caregivers reported that the whole activity was a boost to their self-esteem because participants were not only part of something that was a manifestation of interest by some, but also because their opinion was valued. Accordingly, they considered the experience a well-established assistive technology for senior citizens.

Following such statements, in addition to camera recordings and field notes, it was possible to demonstrate that participants had a positive experience with 360° VR as assistive technology. Overall, their comments show enjoyment, nostalgia, and appreciation.

## 5. Discussion

This paper has presented contextual research on the applicability of VR with HMD as an assistive technology for tourism, cycling, and senior citizens’ usage. It proceeded with the complete process of structuring and performing a 360° VR cyclotouristic experience, and the main results and findings. In accordance with the initial expectation of the possibility that 360° VR could be a solution for assisting senior cyclotourism, it was possible to answer the research question “How can 360° VR foster senior citizens’ cyclotourism experience?”. By cross-referencing the literature with the participants’ opinions, it was found that the experiment was a reliable tool to promote an active lifestyle while encouraging a safe way to perform (cyclo) tourism and increase social behaviors.

Based on the literature review, 360° VR tourism has the potential to be a resource to represent and immerse participants into real environments, whilst stimulating entertainment and engagement, sustainability, low-cost solutions, and information sharing. Such reports were noticeable in this study’s experiment because it enabled senior citizens to impersonate cyclotourists that travel and experience some of Paris, without physically leaving the laboratory site. Indeed, participants faced not only a sense of independence, but also engagement and immersion that intensified the credibility of the scenario [52]. With the replicability of this experiment, on the one hand, the senior citizens and/or caregivers may have the tools to be able to perform travel planning and management [50], because they have become more informed about Paris, and may lead to a better ratio of expectation and delight. On the other hand, they also contributed, unintentionally, to the decrease in overtourism without depriving themselves of experiencing the city, which is significant to its sustainability and heritage preservation [49].

Regarding the simulation of cyclotourism with a stationary bicycle, it is a simpler form of conducting the experiment and it represents advantages in terms of independence, safety, and control of exercise, without having to be concerned about overcrowded locations, poor pavement conditions, and weather conditions. In fact, it was possible not only to replicate some of the cyclotourist movements within the research experience, such as the pedaling effect generated through the dynamics of the bicycle [60], and to study specific human factors and behaviors having in mind the participants’ characteristics and attitudes towards the experiment [68], but also to perform, unintentionally, physical and mental rehabilitation in a social environment [66]. As mentioned above, some participants had physical impairments and considered the activity to be a good way to exercise and to perform physical therapy, being motivated to join, and perform the experiment, as well as motivating and applauding their peers. 

As for the use of VR by senior citizens, it is alleged that it can promote general wellbeing by benefiting physical, cognitive, and psychosocial functions [77,78], which is associated with the dimensions of assistive technologies (i.e., cognition, emotional, social, and physical state) [70]. It is quite applicable to the context of rehabilitation [77,80], which demonstrates that its combination with cycling can be very positive for this purpose, as previously declared. Likewise, this is a powerful assistive tool that can be used by health care professionals, as confirmed by the recordings and caregivers’ opinions, because it also had a positive impact on the mental health of some participants that were depressed and socially excluded due to being institutionalized and/or to the COVID-19 pandemic. Moreover, the experiment described in this study notably followed the four areas of Campelo and colleagues’ VRehab [80], in (i) beneficiaries and providers—the researchers of this study provided the participants with this technological activity, while senior citizens and their caregivers (when applied) beneficiated without costs; (ii) virtual environment—created with existing 360° videos of Paris; (iii) social engagement—visible in attitude change, social interactions, and sense of immersion; and (iv) healthcare application—in the form of assistive technology for physical, cognitive, and psychosocial therapy. 

In terms of results, it was possible to corroborate both hypotheses: (a) there are differences in younger and older senior citizens concerning the promotion of the touristic experience; and (b) social interaction and safety confidence in VR activities are key for senior citizens to have fun. In detail, even if, broadly, the experiment was considered good to foster senior cyclotourism, older participants tended to give greater importance to the fact that their friends, family, and caregivers encouraged them to use 360° VR, which contributed to the way they feel about the suitability of it to perform (cyclo) tourism to know places and exercise, share the experience, and use it in the future. Additionally, such encouragement may be seen as a strategy for social interactions and a sense of assuredness in using the technology and having fun with it.

A stronger correlation of Q1 and Q2 with any other variables was expected, because it was easy to assume that visibility and comfort would affect—positively if visible and comfortable and negatively if not—the experience significantly; however, it did not occur. A positive correlation between Q3 and Q4 was expected; however, participants tended to find the experiment more enjoyable than fun. It was not anticipated that fun would have an association with VR being good to conduct tourism instead of correlating with sharing the activity. It was also predicted that if family and friends influenced participants, they would like to share with them (Q5 and Q13), but it was not anticipated that influence was so positive in the participants’ perception of VR being good (cyclo)tourism and safe exercise. In the same vein, it was considered that Q7 and Q8 would be correlated; however, this was not demonstrated. Q9 had the most correlations, which was also expected because VR seems to be a suitable tool to perform tourism. Nonetheless, the several positive correlations on the use of technology in cyclotourism safeness to know places, exercise, and socialize surpass the expectations.

Thereby, the study reported in this paper corroborates the literature and related studies on VR tourism, cycling, and usage by senior citizens, having expected and unexpected results and findings. It set out to overcome the fact that 360° VR with HMD as an assistive technology applied to senior cyclotourism may not be deeply studied in academia, and it could be a valuable contribution to social science studies within wellbeing and behavior change towards an active and healthy ageing promotion. Furthermore, this research addresses the effectiveness of 360° VR with HMD and a simple setup to adhere to cyclotourism and foster safe conviviality between different profiles of senior citizens (i.e., different age cohorts and backgrounds). Thus, it is possible to assume that 360° VR can foster cyclotourism due to its proximity to reality, its tendency to be an enjoyable and a fun technology, and its comfort when used in short periods (since the testing was performed at 3-minute intervals). It also may serve as an assistive technology for senior citizens and disabled people not only to carry out tourism, but also as a rehabilitation and social interaction tool.

## 6. Conclusions

The background research about VR evidences the potential of improving senior citizens’ wellbeing by promoting socially engaged states. However, even if VR studies have been progressively gaining interest and awareness—because it demonstrates possibilities for impacting human behavior, contributing to cognitive health, and for rehabilitation by simulating reality situations and/or tasks by providing greater motivation—assisting senior citizens’ independence, rehabilitating functional capabilities, and improving emotional and social wellbeing remain areas to be explored.

In a nutshell, this study further extends the current knowledge on VR research by applying it to a new subject (assisting senior cyclotourism), providing positive affective-motivational states and satisfaction in 360° VR with HMD assistive experiment. It counts not only on the participants’ feedback, but also on their caregivers’ (when applied) opinions on the use of this experiment as an assistive technology, which seems able to aid cognitive, social, emotional, and physical improvement. It also contributes to tourism and active and healthy ageing research by empirically showing the reactions of senior citizens using VR technology, emphasizing their enjoyment, engagement and social interactions, and interest in cyclotourism. Additionally, the empirical methodology may allow a reliable and simple setup for different application subjects (e.g., by using a treadmill instead of a stationary bicycle for indoor tourism and/or exercise), decreasing the possible limitations related to experience customization, and surpassing the constraints of previous studies of VR and 360° VR.

It is important to acknowledge the consideration of a few limitations for this research; therefore, the results must be interpreted with caution. Firstly, the subject of VR and 360° VR in the context of assistive technology in cyclotourism for active and healthy ageing is an unexplored topic, with the experience performed in this study being pioneering research. Secondly, a convenience sample was recruited; hence, attempts to generalize beyond these participants are not justified because the outcomes are not warranted. Thirdly, there may be some respondents’ bias in the results obtained, because each group of participants was from the same institute or were acquaintances. However, the researchers tried to ensure that all participants expressed their opinions of the experience, interpreting the outcomes based on the questionnaire’s responses and camera records. Finally, this study was performed during the COVID-19 outbreak, having the need to shorten the activity to comply with all safety and hygiene measures. This fact may have influenced the pragmatism of the experience, not allowing for greater social moments that could have improved its results.

Regarding future work, a longitudinal study with longer periods of testing would be beneficial to sustain the obtained results; however, it is important to gather the conditions that allow the research to be carried out safely (concerning the pandemic and the target audience). Supplementary research needs to be carried out in the psychology field, to understand the use of VR as an assistive tool with other motivational technologies, such as gamification, while analyzing whether there is an impact on behavior change towards active and healthy ageing, as well as in the use and frequency of cyclotourism indoors and outdoors. Moreover, adding the VR component to exergames (e.g., Zwift) or current cyclotourism and cycling apps would be beneficial to this research and probably a significant contribution to senior citizens, disabled and institutionalized people, and/or people incapable of riding a bicycle outside due to the pandemic, or psychological and/or physical limitations. Lastly, it is intended to improve the experience and incorporate gamification elements to foster storytelling, competition, and cooperation behaviors, as well as explore and analyze the immersion of senses in a profound way.

## Figures and Tables

**Table 1:** Description of VR cyclotourism experience phases.

	Duration	Main Equipment	Activity Purpose
**Phase 1**	3 minutes per participant	Armchair, HMD	Adaptation to 360° VR and HMD
**Phase 2**	3.5 minutes per participant	Stationary bicycle, HMD	360° VR cyclotouristic experience

**Table 2:** Overview of the items from the questionnaire and correspondent questions for data analysis.

Data Collection Items	Data Analysis Questions
Q1. The virtual reality experience was easy to see.	What are the main aspects that affect participants’ condition of vision?
Q2. The virtual reality experience was comfortable (e.g., no nausea).	What are the main aspects that influence participants’ discomfort and/or sickness?
Q3. I enjoyed using virtual reality technology.	Is VR a useful tool for entertainment?
Q4. I had fun using virtual reality technology.	
Q5. My family and friends influenced me to use virtual reality.	Are participants influenced by people close to them?
Q6. My caregivers encouraged me to use virtual reality.	
Q7. Virtual reality can be a technology capable of improving my state of mind.	Is VR a useful tool for wellbeing improvement?
Q8. In the future, I would like to use virtual reality to relax my mind.	
Q9. The virtual reality experience is good to do tourism.	Which are the main features that a VR experience should have to be closed to tourism?
Q10. The experience in virtual reality, combined with cyclotourism, is a good way to get to know places in a safe way.	What are the main factors of using VR in cyclotourism that contribute to active and healthy ageing?
Q11. The virtual reality experience on the bike is a good way to exercise in a safe way.	
Q12. The experience in virtual reality with a bicycle was a good time for socializing.	Can VR enhance social interactions?
Q13. The virtual reality experience makes me want to share it with my friends and family.	

**Table 3:** Demographic characterization of the participants.

Gender	*n*	%
Female	59	78
Male	17	22
Total	76	100
**Age cohorts**	***n***	**%**
Pre-Senior (50–64)	16	21.1
Young-Senior (65–74)	20	26.3
Middle-Senior (75–84)	20	26.3
Old-Senior (85+)	20	26.3
Total	76	100

**Table 4:** Global results from the questionnaire.

	1 I Totally Disagree		2 I Disagree		3 Neutral		4 I Agree		5 I Totally Agree	
	*n*	%	*n*	%	*n*	%	*n*	%	*n*	%
**Q1**	0	0	2	2.6	1	1.3	3	3.9	70	92.1
**Q2**	0	0	3	3.9	2	2.6	6	7.9	65	85.5
**Q3**	0	0	0	0	0	0	2	2.6	74	97.4
**Q4**	0	0	0	0	0	0	5	6.6	71	93.4
**Q5**	0	0	1	1.3	5	6.6	2	2.6	65	85.5
**Q6**	0	0	0	0	3	3.9	0	0	66	86.8
**Q7**	0	0	1	1.3	5	6.6	5	6.6	65	85.5
**Q8**	1	1.3	1	1.3	2	2.6	7	9.2	65	85.5
**Q9**	0	0	0	0	1	1.3	5	6.6	70	92.1
**Q10**	0	0	0	0	4	5.3	5	6.6	67	88.2
**Q11**	0	0	2	2.6	1	1.3	4	5.3	69	90.8
**Q12**	0	0	0	0	0	0	4	5.3	72	94.7
**Q13**	0	0	2	2.6	3	3.9	2	2.6	69	90.8

**Table 5:** Pearson’s correlation between age and influence of others on using VR (Q5, Q6); in using VR in the future (Q8); being good to do tourism (Q9); being adequate to know places and exercise safely (Q10, Q11); sharing the experience (Q13).

	Q5	Q6	Q8	Q9	Q10	Q11	Q13
**Age**	r = 0.474 **	r = 0.254 *	r = 0.330 **	r = 0.350 **	r = 0.230 *	r = 0.426 **	r = 0.488 **

**Table 6:** Significant Pearson’s correlation results between each question from the questionnaire.

**r**	**Q2**	**Q3**	**Q4**							
**Q1**	0.489 **	0.401 **	0.313 **							
**r**	**Q1**	**Q3**	**Q4**	**Q8**	**Q11**	**Q13**				
**Q2**	0.489 **	0.298 **	0.288 **	0.244 *	0.237 *	0.291 **				
r	**Q1**	**Q2**	**Q4**	**Q7**	**Q12**					
**Q3**	0.401 **	0.298 **	0.620 **	0.471 **	0.329 **					
r	**Q1**	**Q2**	**Q3**	**Q7**	**Q8**	**Q9**	**Q10**	**Q11**	**Q12**	**Q13**
**Q4**	0.313 **	0.288 **	0.620 **	0.417 **	0.268 *	0.567 **	0.443 **	0.397 **	0.651 **	0.348 **
r	**Q6**	**Q8**	**Q9**	**Q11**	**Q12**	**Q13**				
**Q5**	1.000 **	0.485 **	0.537 **	0.695 **	0.266 *	0.799 **				
r	**Q5**	**Q11**	**Q13**							
**Q6**	1.000 **	0.387 **	0.768 **							
r	**Q3**	**Q4**	**Q8**	**Q9**	**Q11**	**Q12**	**Q13**			
**Q7**	0.471 **	0.417 **	0.321 **	0.475 **	0.388 **	0.390 **	0.267 *			
r	**Q2**	**Q4**	**Q5**	**Q7**	**Q9**	**Q11**	**Q12**	**Q13**		
**Q8**	0.244 *	0.268 *	0.485 **	0.321 **	0.531 **	0.611 **	0.231 *	0.648 **		
r	**Q4**	**Q5**	**Q7**	**Q8**	**Q10**	**Q11**	**Q12**	**Q13**		
**Q9**	0.567 **	0.537 **	0.475 **	0.531 **	0.543 **	0.909 **	0.823 **	0.683 **		
r	**Q4**	**Q9**	**Q11**	**Q12**	**Q13**					
**Q10**	0.443 **	0.543 **	0.421 **	0.512 **	0.366 **					
r	**Q2**	**Q4**	**Q5**	**Q6**	**Q7**	**Q8**	**Q9**	**Q10**	**Q12**	**Q13**
**Q11**	0.237 *	0.397 **	0.695 **	0.387 **	0.388 **	0.611 **	0.909 **	0.421 **	0.666 **	0.818 **
r	**Q3**	**Q4**	**Q5**	**Q7**	**Q8**	**Q9**	**Q10**	**Q11**	**Q13**	
**Q12**	0.421 **	0.651 **	0.266 *	0.390 **	0.231 *	0.823 **	0.512 **	0.666 **	0.309 **	

## Data Availability

Not applicable.
